# Huaier Cream Protects against Adriamycin-Induced Nephropathy by Restoring Mitochondrial Function via PGC-1**α** Upregulation

**DOI:** 10.1155/2015/720383

**Published:** 2015-03-11

**Authors:** Ruochen Che, Chunhua Zhu, Guixia Ding, Min Zhao, Mi Bai, Zhanjun Jia, Aihua Zhang, Songming Huang

**Affiliations:** ^1^Department of Nephrology, Nanjing Children's Hospital, Nanjing Medical University, 72 Guangzhou Road, Nanjing, Jiangsu 210029, China; ^2^Institute of Pediatrics, Nanjing Medical University, Nanjing, Jiangsu 210008, China

## Abstract

The mechanism by which Huaier, a Chinese traditional medicine, protects podocytes remains unclear. We designed the present study to examine whether mitochondrial function restored by PGC-1*α* serves as the major target of Huaier cream in protecting ADR nephropathy. After ADR administration, the podocytes exhibited remarkable cell injury and mitochondrial dysfunction. Additionally, ADR also reduced PGC-1*α* both *in vivo* and *in vitro*. Following the Huaier treatment, the notable downregulation of PGC-1*α* and its downstream molecule mitochondrial transcription factor A (TFAM) were almost entirely blocked. Correspondingly, Huaier markedly ameliorated ADR-induced podocyte injury and mitochondrial dysfunction in both rat kidneys and incubated cells as it inhibited the decrease of nephrin and podocin expression, mtDNA copy number, MMP, and ATP content. Transmission electron microscopy result also showed that Huaier protected mitochondria against ADR-induced severe mitophagy and abnormal changes of ultrastructural morphology. In conclusion, Huaier can protect podocytes against ADR-induced cytotoxicity possibly by reversing the dysfunction of mitochondria via PGC-1*α* overexpression, which may be a novel therapeutic drug target in glomerular diseases.

## 1. Introduction

Nephrotic syndrome, characterized by massive proteinuria, hypoalbuminemia, peripheral edema, and hyperlipidemia, is one of the most common kidney diseases in children and adolescents [1]. Although glucocorticoids (GCs), the mainstay of therapy for over 50 years, are effective in most children, more than 20% develop GC-resistant nephrotic syndrome [2]. Additionally, most children who are sensitive to GCs will experience the relapse, among which 50% will develop frequently relapsing or steroid-dependent nephrotic syndrome [3]. So combined therapy with other immunosuppressors has to be implemented. Almost all the alternative treatments we usually use today, like cyclosporine A and cyclophosphamide, have some serious adverse effects. Cyclosporine A can induce gingival hyperplasia, hirsutism, and, the most concentrated side effects to nephrologists, nephrotoxicity [4]. The side effects of cyclophosphamide include nausea, infection, bone marrow suppression, and alopecia, of which the frequently worried about problems are possibilities of sterility and carcinoma. Given the unsatisfactory situation about nephrotic syndrome treatment, nephrologists are eager to find promising novel therapies of nephrotic syndrome. Podocyte injury plays a critical role in GC resistance in idiopathic nephrotic syndrome. Particularly in focal segmental glomerular sclerosis (FSGS), a frequent cause of end-stage renal disease with a prevalence of 4% in USA, podocytes are at significantly increased risk of injury and loss, a phenomenon called podocytopathy [5]. When podocytes are damaged, nephrotic proteinuria occurs following effacement of the podocyte foot processes and rearrangement of the actin cytoskeleton. This discovery has advanced the field of podocyte-targeted therapies.

Nowadays Chinese traditional herbs targeting nephropathy have drawn much attention [6–8]. Huaier cream, a Chinese traditional medicine, is a kind of* Trametes robiniophila* Murr., of which the extract has potent free radical-scavenging and immunomodulating properties [9]. We previously reported that Huaier exerts a protective effect in the adriamycin (ADR) nephropathy rat model by inhibiting inflammatory cytokine expression and preventing podocyte injury [10]. The proteinuria of ADR-induced rats was significantly decreased with Huaier treatments and fusion of podocyte processes was alleviated [10]. Although the protective effect of Huaier on ADR nephropathy is very impressive, the molecular mechanism remains elusive.

Growing evidences have shown the important role of mitochondrial dysfunction in mediating the podocyte injury [11–13]. Peroxisome proliferator-activated receptor-*γ* coactivator 1*α* (PGC-1*α*) has been shown as a critical regulator of mitochondrial function in podocytes [14]. Our recent findings also demonstrated that ADR remarkably suppressed the PGC1-*α* expression in podocytes paralleled with disrupted mitochondrial function and podocytes injury. Restoration of PGC-1*α* by adenovirus-based gene transfer markedly improved mitochondria dysfunction, abolished ROS production, and attenuated podocytes injury following ADR treatment [15]. Based on our findings described above, we speculated that Huaier may protect against podocyte injury in glomerular disease via maintaining PGC-1*α* expression and, consequently, mitochondrial function.

Animal model of ADR nephropathy is widely used as an analogue of human FSGS showing the reduced renal function, proteinuria, podocyte dysfunction, and tubulointerstitial fibrosis [16]. The findings from ADR nephropathy model may be closely correlated to the pathogenesis and therapy of FSGS in clinic. Therefore, the striking efficacy of Huaier in treating ADR nephropathy and its underlying mechanism might be applicable to human FSGS.

## 2. Materials and Methods

### 2.1. Reagents

Huaier cream, the hot water extract of* Trametes robiniophila* Murr., was bought from Gaitianli Pharmaceutical Co. (Qidong, Jiangsu Province, China). Huaier cream is a complex of polysaccharide, proteins, and mineral substances, of which 95% is the effective substance, polysaccharide. Polysaccharide, 30,000D, is soluble in water. The PH of aqueous solution is 5-6. Caelyx (liposomal ADR) was purchased from Merck (Whitehouse Station, NJ). Anti-cytochrome *c* antibody and 2′,7′-dichlorofluorescein diacetate (DCFDA) were purchased from Sigma (St. Louis, MO). We used anti-nephrin, anti-podocin (Abcam, Cambridge, MA), anti-PGC-1*α* (Santa Cruz Biotechnology, Santa Cruz, CA), anti-COX IV, and anti-*β*-actin antibodies (Cell Signaling Technology, Beverly, MA). SYBR Green master mix for real-time PCR was provided by Applied Biosystems (Foster City, CA).

### 2.2. Animals

All experiments were performed with the approval of the experimental animal committee of Nanjing Medical University. Male Sprague-Dawley rats (180 ± 15 g) were bought from Shanghai SLAC Laboratory Animals Co., Ltd. (Shanghai, China). The animals were housed and fed under standard conditions. The rats were randomly assigned to three treatment groups (*n* = 6 each): the ADR group received a single, slow injection of 5 mg/kg ADR; 1 day after treatment with ADR, the Huaier-treated (Huaier + ADR) group was further treated with a daily oral gavage of 2 mg/kg Huaier cream for 15 days; the control rats received injections and oral gavages of pure saline. At the end of the experiment, rats were anesthetized with 5 mg/kg urethane. The serum was isolated and the kidneys were harvested.

### 2.3. Cell Culture and Treatments

MPC5 conditionally immortalized mouse podocyte clonal cells (kindly provided by Peter Mundel at Mount Sinai School of Medicine through Dr. Jie Ding at Peking University) were cultured and induced to differentiate as described [17]. Briefly, podocytes were maintained with interferon-*γ* at 33°C for proliferation and were cultured without interferon-*γ* at 37°C to induce differentiation. All cells were between passages 3 and 5.

Before experiments, the cells were subcultured to 80–90% confluency in various sizes of culture vessels (six-well plates, 60 mm dishes), depending on the number of cells required by the protocols, and were then incubated in 1% fetal bovine serum-supplemented medium for 24 h to make the cells quiescent. To determine the proper stimulatory dose, we tried ADR concentrations from 50 nM to 200 nM and finally chose 200 nM for use in experiments. To explore whether Huaier protects podocyte mitochondria, we pretreated cells with Huaier (0.2 mg/mL) for 1 h before 200 nM ADR stimulation. The duration of stimulation was based on the protocol requirements, which are illustrated for each experiment.

### 2.4. Podocyte Apoptosis

After treatment, Annexin V-fluorescein isothiocyanate and propidium iodide double staining (Annexin V: fluorescein isothiocyanate apoptosis detection kit, BD Biosciences, San Diego, CA) were used to stain the podocytes following the manufacturer's protocol. We quantified apoptosis by flow cytometry (Becton, Dickinson and Company, USA).

### 2.5. PCR

Total RNA from cultured podocytes and renal cortex was isolated using the TRIzol total RNA isolation kit (Invitrogen, Carlsbad, CA). Single-stranded cDNAs were obtained by reverse transcription following the manufacturer's protocol. Total DNA from cultured podocytes and renal cortex was isolated using the DNeasy Tissue Kit (Invitrogen, Carlsbad, CA). The sequences of the primer pairs were in Tables 1 and 2. The mRNA and mtDNA copy numbers were detected by real-time PCR. Amplification was performed using the ABI 7300 real-time PCR detection system (Foster City, CA) with SYBR Green master mix (Applied Biosystems, Foster City, CA). Glyceraldehyde 3-phosphate dehydrogenase (GAPDH) and 18S rRNA served as internal controls of mRNA and mtDNA, respectively. The thermal cycling conditions were 95°C for 10 min, followed by 40 cycles of 95°C for 15 s and 60°C for 1 min.

### 2.6. Reactive Oxygen Species (ROS) and Mitochondrial Superoxide Measurement

DCFDA is a cell-permeable fluorogenic dye that can be oxidized by H_2_O_2_ to produce fluorescence and is therefore used to monitor intracellular generation of ROS as previously described [18]. To measure ROS, podocytes were cultured in six-well plates until confluence. Plates were incubated with 10 *μ*M DCFDA in the dark at 37°C for 30 min. Then the podocytes were harvested and washed twice with Hanks' balanced salt solution (Sigma, St. Louis, MO), and the fluorescence was measured by flow cytometry.

The ROS generated by mitochondria were measured by MitoSOX Red reagent (Invitrogen, Carlsbad, CA), an indicator of mitochondrial superoxide. Briefly, 1 mL MitoSOX Red reagent working solution was incubated with podocytes adhering to the six-well plates at 37°C in the dark at a final concentration of 5 *μ*M for 10 min. Then the fluorescence was measured as above.

### 2.7. Mitochondrial Membrane Potential (MMP)

Podocyte MMP was monitored by JC-1, which is a MMP-sensitive fluorescent dye as previously described [19]. The prepared dissociated podocytes were incubated in the dark with JC-1 (7.5 mM, 30 min at 37°C). Then the cells were washed with JC-1 washing buffer precooled at 4°C, and fluorescence was detected by flow cytometry. The relative MMP was calculated using the ratio of J-aggregate to monomer (590/520 nm). Values are expressed as the fold increase in J-aggregate/monomer fluorescence over control cells. To obtain a direct image of the fluorescence change, the podocytes were grown on glass cover slips. After treatment, the podocytes were stained by JC-1 and examined by confocal microscopy.

### 2.8. ATP Measurement

ATP in podocytes and renal cortex was detected with a luciferase-based bioluminescence assay kit (Sigma-Aldrich, St. Louis, MO) in a FLUOstar Optima reader following the manufacturer's instructions. Each total ATP level was calculated as luminescence normalized to protein concentration.

### 2.9. Western Blot

Podocytes or renal cortex homogenate (100 mg) was lysed in protein lysis buffer with protease inhibitor cocktail (Sigma, St. Louis, MO) for 20 min on ice. After centrifugation, the supernatant was harvested, and the lysate protein concentrations were measured by a BCA Protein Assay Kit (Pierce). Immunoblotting was performed with anti-nephrin (1 : 200), anti-podocin (1 : 500), anti-PGC-1*α* (1 : 200), or anti-*β*-actin antibody (1 : 1000), followed by horseradish peroxidase-labeled secondary antibodies and ECL visualization. Bands were visualized by a GS-800 calibrated densitometer (Bio-Rad, Philadelphia, PA), and densitometry was performed by Quantity One software (Bio-Rad, Philadelphia, PA).

### 2.10. Measurement of Cytochrome *c* Release from Mitochondria to Cytoplasm

To isolate the mitochondria and cytoplasm, renal cortex was washed, homogenized, and prepared with a Mitochondria Isolation Kit for Tissue (Pierce, Rockford, IL) following the manufacturer's instructions. Western blotting was performed to detect cytochrome *c* in mitochondria and cytoplasm. COX IV (1 : 500) and *β*-actin (1 : 1000) were used as the internal reference protein for mitochondria or cytoplasm, respectively.

### 2.11. Transmission Electron Microscopy

To evaluate mitochondrial morphology, live podocytes were collected, fixed in 1.25% glutaraldehyde/0.1 M phosphate buffer, and postfixed in 1% OsO_4_/0.1 M phosphate buffer. Ultrathin sections (60 nm) were cut on a microtome, placed on copper grids, stained with uranyl acetate and lead citrate, and examined in an electron microscope (JEOL JEM-1010, Tokyo, Japan).

### 2.12. Statistical Analysis

All results are presented as mean ± SD (standard deviation). The statistical analysis was done by SPSS 16.0 using one-way analysis of variance. A *P* value of less than 0.05 was considered statistically significant.

## 3. Results

### 3.1. Huaier Blocked ADR-Induced Podocyte Injury* In Vitro*

**Figure 1:**
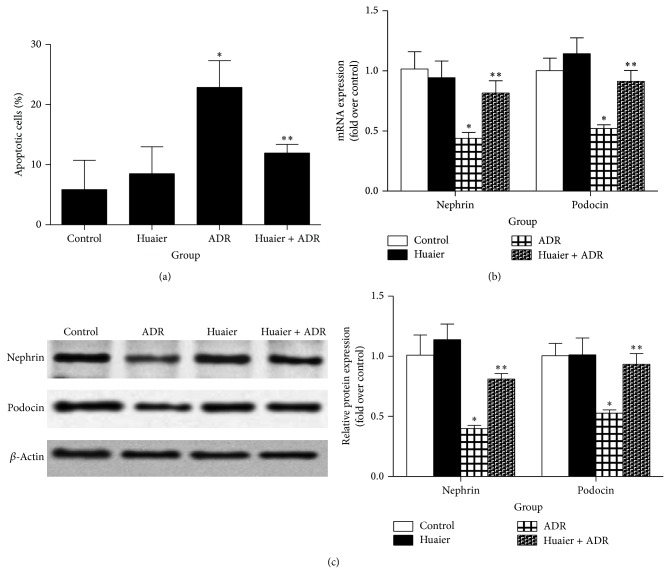
Huaier protected podocytes against ADR-induced injury* in vitro*. (a) Percentage of apoptotic cells. (b) Real-time RT-PCR of nephrin and podocin. (c) Western blot of nephrin and podocin. Left: representative immunoblots. Right: densitometric analysis. Podocytes were pretreated with Huaier (0.2 mg/mL) for 1 h followed by incubation with ADR (200 nM) for further 48 h (for apoptosis analysis, (a)) or 24 h (for real-time RT-PCR analysis (b); immunoblotting analysis (c)). Data are expressed as the means ± SD (*n* = 6). ^*^ *P* < 0.05 versus control. ^**^ *P* < 0.05 versus ADR group.

We investigated whether Huaier could protect podocytes against ADR-induced injury* in vitro*. ADR caused striking podocytes apoptosis in line with the significant downregulation of nephrin and podocin at both mRNA and protein levels (Figures 1(a)–1(c)). Huaier administration remarkably decreased the percentage of apoptotic cells and strikingly alleviated the ADR-induced downregulation of nephrin and podocin (Figures 1(a)–1(c)).

### 3.2. Huaier Blocked ADR-Induced Mitochondrial Dysfunction* In Vitro*

**Figure 2:**
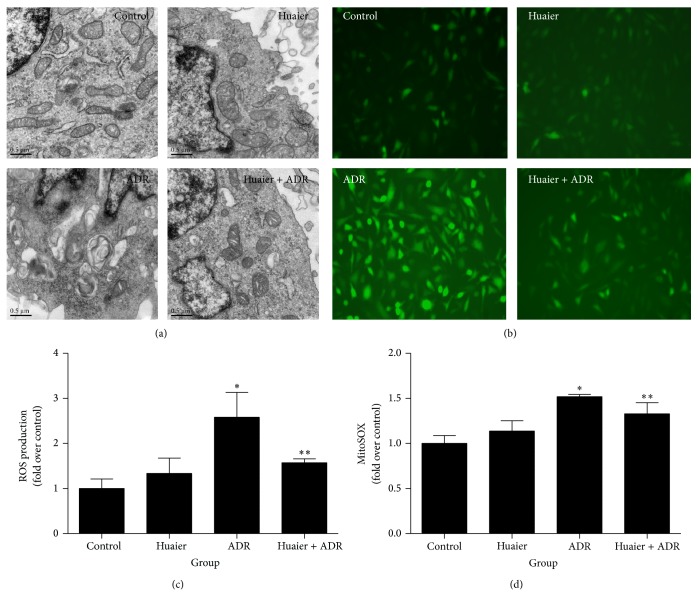
Huaier blocked ADR-induced mitochondrial dysfunction* in vitro*. (a) Mitochondrial morphology imaged by transmission electron microscopy. (b) Representative images of podocytes stained with dichlorodihydrofluorescein diacetate (DCFDA). (c) Quantitation of 2′,7′-dichlorofluorescein (DCF) fluorescence by flow cytometry. (d) Quantitation of MitoSOX fluorescence by flow cytometry. Podocytes were pretreated with Huaier (0.2 mg/mL) for 1 h followed by incubation with ADR (200 nM) for further 24 h. Data are expressed as the means ± SD (*n* = 6). ^*^ *P* < 0.05 versus control. ^**^ *P* < 0.05 versus ADR group.

**Figure 3:**
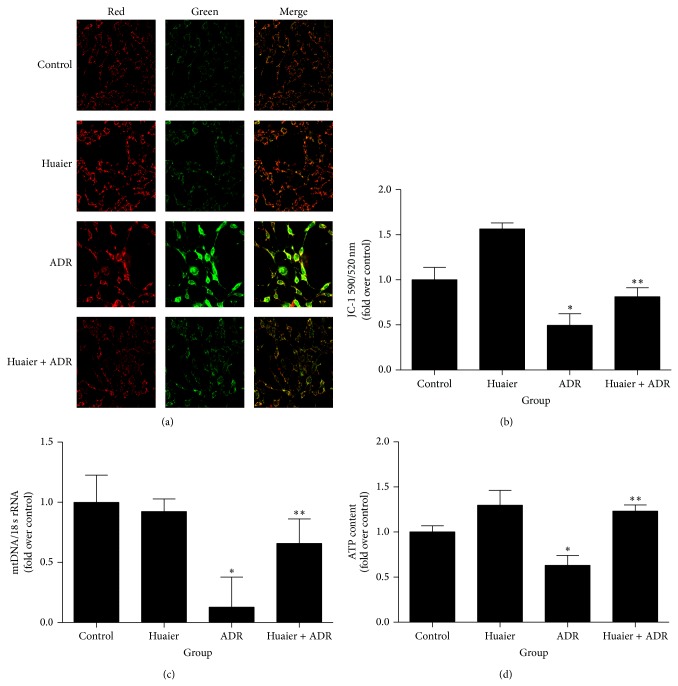
Huaier blocked ADR-induced mitochondrial dysfunction* in vitro*. (a) Representative images of podocytes stained with JC-1. (b) MMP measurements by flow cytometry. (c) Real-time RT-PCR analysis of mtDNA copy number. (d) ATP content. Podocytes were pretreated with Huaier (0.2 mg/mL) for 1 h followed by incubation with ADR (200 nM) for further 24 h. Data are expressed as the means ± SD (*n* = 6). ^*^ *P* < 0.05 versus control. ^**^ *P* < 0.05 versus ADR group.

Then we detected various parameters of mitochondrial function* in vitro*, including mitochondrial ROS production, mtDNA copy number, ATP content, MMP, and mitochondrial morphology. MitoSOX Red was used to measure mitochondria-derived ROS. All the mitochondrial functional parameters were disrupted by ADR as shown by reduction of mtDNA copy number and ATP content, abnormality of MMP, and overproduction of mitochondria-derived ROS. Intriguingly, such disruptions caused by ADR were robustly attenuated by Huaier (Figures 2 and 3). By transmission electron microscopy, we observed severe mitophagy and mitochondrial swelling with fracture and fusion of cristae in the ADR group. In contrast, Huaier-pretreated cells exhibited largely normal mitochondria morphology (Figure 2(a)).

### 3.3. Huaier Ameliorated ADR-Induced Mitochondrial Dysfunction in Rat Kidneys

**Figure 4:**
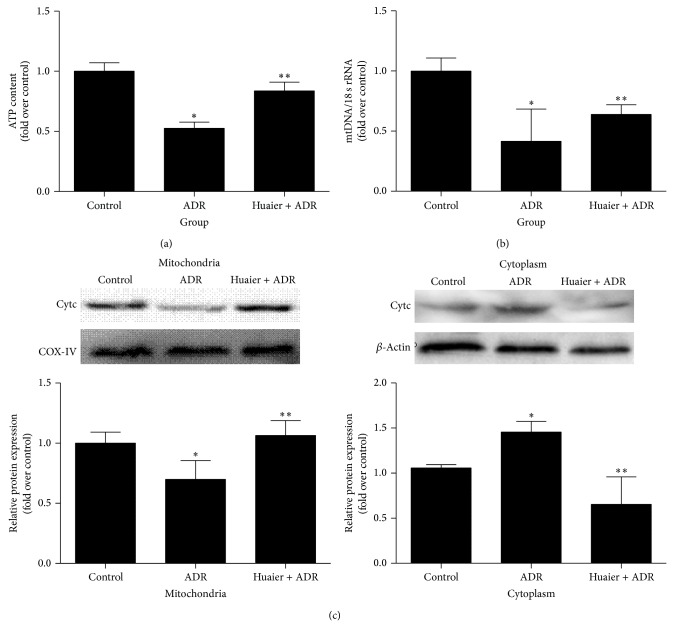
Huaier ameliorated ADR-induced mitochondrial dysfunction in rats. (a) ATP content. (b) Real-time PCR analysis of mtDNA copy number. (c) Western blot analysis of mitochondrial and cytosolic cytochrome *c*. Upper: representative immunoblots. Lower: densitometric analysis. Data are expressed as the means ± SD (*n* = 6). ^*^ *P* < 0.05 versus control. ^**^ *P* < 0.05 versus ADR group.

Our previous report had shown that Huaier significantly reduced proteinuria and prevented podocyte injury in ADR-treated rats. The kidney function was also examined. Blood urea nitrogen and serum creatinine of all the three groups were within normal range without statistical significance [10]. To investigate whether Huaier protects against ADR-indued mitochondria dysfunction* in vivo*, we measured ATP production, quantity of mtDNA, and cytochrome *c* release in ADR-treated rat kidneys with or without Huaier (Figures 4(a)–4(c)). As expected, the ADR-treated group exhibited reduced ATP production and decreased mitochondrial copy number in the renal cortex, which was markedly alleviated by Huaier treatment. Once the defense against ADR cytotoxicity fails, the MMP decreases and the mitochondrial outer-membrane pores open. Then cytochrome *c* is released from mitochondria into the cytoplasm [20, 21]. We observed that ADR increased the cytochrome *c* release into the cytoplasm, which was abolished by Huaier administration, indicating a maintained normal MMP (Figure 4(c)).

### 3.4. Huaier Upregulated PGC-1**α** Expression* In Vivo*

**Figure 5:**
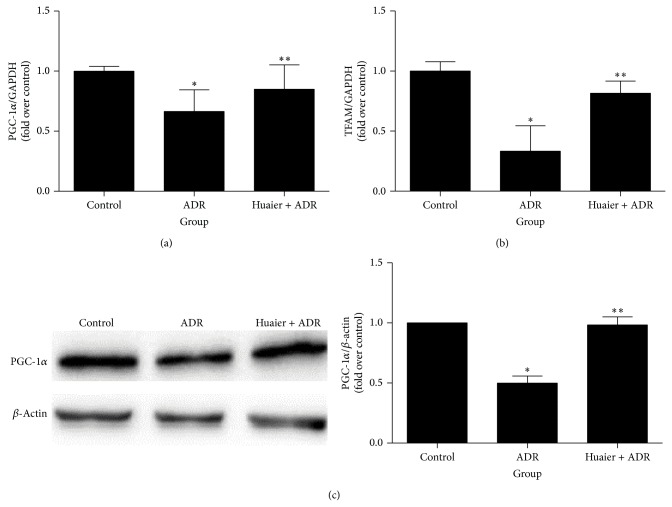
Huaier upregulated PGC-1*α in vivo*. (a) Real-time RT-PCR of PGC-1*α*. (b) Real-time RT-PCR of TFAM. (c) Western blot of PGC-1*α*. Left: representative immunoblots. Right: densitometric analysis. Data are expressed as the means ± SD (*n* = 6). ^*^ *P* < 0.05 versus control. ^**^ *P* < 0.05 versus ADR group.

To further test our hypothesis, we measured PGC-1*α* expression in ADR-treated rat kidneys (Figure 5). As shown by the results, ADR significantly reduced the PGC-1*α* expression at both mRNA and protein levels. Meanwhile, mitochondrial transcription factor A (TFAM), a downstream molecular target of PGC-1*α*, was also dramatically decreased as determined by quantitative real-time PCR following ADR administration. Such alterations were almost completely abolished by Huaier treatment (Figure 5). These results importantly suggested that Huaier restores mitochondrial function possibly through upregulating PGC-1*α*, a critical mechanism mediating ADR-induced podocytes injury.

### 3.5. Huaier Upregulated PGC-1*α* Expression* In Vitro*

**Figure 6:**
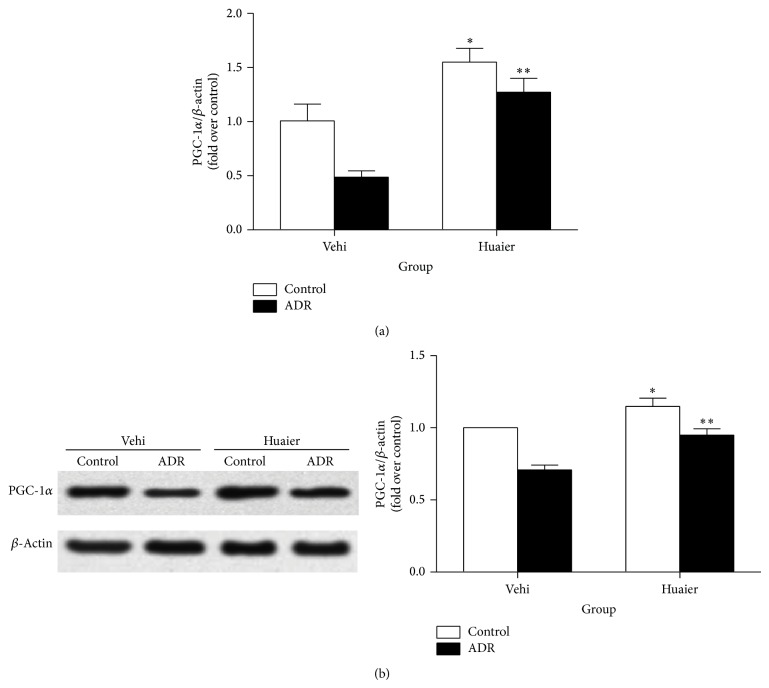
Huaier induced PGC-1*α* expression* in vitro*. (a) Real-time RT-PCR of PGC-1*α*. (b) Western blot of PGC-1*α*. Left: representative immunoblots. Right: densitometric analysis. Podocytes were pretreated with Huaier (0.2 mg/mL) for 1 h followed by incubation with ADR (200 nM) for further 24 h. Data are expressed as the means ± SD (*n* = 6). ^*^ *P* < 0.05 control of Huaier group versus control of vehicle group. ^**^ *P* < 0.05 versus ADR of Huaier group versus ADR of vehicle group.

We further examined the Huaier effect on PGC-1*α* regulation* in vitro*. As shown by the data (Figure 6), ADR-induced PGC-1*α* reduction can be restored by Huaier in podocytes at both mRNA and protein levels, which indicated a direct effect of Huaier on opposing the ADR-induced PGC-1*α* downregulation in podocytes.

## 4. Discussion

Our previous study demonstrated that ADR induced podocyte injury via disrupting PGC-1*α* expression and mitochondrial function [15]. We also reported that Huaier cream, a traditional Chinese herb, protected against ADR-induced nephropathy in rat with unclear mechanisms. Here we presented the new finding indicating that mitochondrial function protected by PGC-1*α* upregulation serves as the major target of Huaier cream and might be responsible for the beneficial effect of Huaier against ADR-induced podocyte injury.

Although ADR is widely used for the treatment of solid and hematogenous tumors, the high-risk side effects including ADR-related nephropathy and cardiomyopathy significantly limited its application in patients. In animal studies, ADR nephropathy is a classic model mimicking the pathology of human FSGS. ADR chiefly acts on the podocytes and interrupts the integrity of glomerular filtration barrier, which subsequently results in the proteinuria and nephrotic syndrome [16]. The podocytes injury and consequent podocytes depletion may finally result in the glomerulosclerosis [22]. Although the ADR related tissue injury received extensive studies, the mechanisms are still less understood. Lahoti et al. reported that ADR can induce mitochondrial perturbations and activate cell death genes in kidney [23]. In agreement with TS's report, we also observed that ADR caused mitochondria dysfunction and cell apoptosis in ADR-treated podocytes and kidneys [15]. And such a dysfunction of mitochondria could be responsible for ADR toxicity in kidney cells to some extent.

In the present study, Huaier treatment strikingly attenuated the podocyte injury and mitochondrial dysfunction caused by ADR* in vivo* and* in vitro*. It is well known that the injured mitochondria produce excessive intracellular ROS which further cause the damage of organelles and the decrease of ATP production due to the oxidation of mitochondrial thiols [24, 25]. The damaged mitochondria have increased permeability which allows the release of cytotoxic and proinflammatory substances, such as cytochrome *c* and mtDNA, to start the proapoptotic cascade [20, 21]. In agreement with this theory, we found that the mitochondria disruption in ADR-treated cells or kidneys was accompanied with high levels of mitochondria-derived ROS generation and decreased ATP production. Huaier eliminated the ROS overload, restored the redox homeostasis, and protected the cells from apoptosis. Generally, mitochondria dysfunction itself initiated the mitophagy process to remove the damaged mitochondria [26]. By electron microscopy, we observed both ADR-stimulated early-stage mitophagy as shown by autophagosomes with engulfed mitochondria and late-stage mitophagy as shown by single-membrane autolysosomes with the same electron density of residual mitochondria. In the Huaier plus ADR group, Huaier restrained mitochondrial depolarization, although ADR still caused a certain degree of mitochondrial swelling and deformation.

PGC-1*α* has an established role in maintaining the normal mitochondrial function. Convincing evidence proved that PGC-1*α* can protect mitochondrial respiratory function and decrease mitochondrial apoptotic susceptibility [14, 27, 28]. Administration of recombinant human mitochondrial transcription factor A, a downstream target of PGC-1*α*, protects the mitochondrial respiration in aged mice [29]. In the present study, ADR downregulated PGC-1*α* and TFAM in both cultured cells and rat kidneys. Huaier maintained the expression of PGC-1*α* and TFAM after ADR stimulation, suggesting a critical role of PGC-1*α* signaling in the mechanism of Huaier action. Collectively, the findings discussed above indicated that Huaier exerted its cytoprotective effects possibly through maintaining the normal mitochondrial function via PGC-1*α* expression. As the coregulator, PGC-1*α* regulates a large number of transcription factors, like PPAR*α*, PPAR*γ*, estrogen receptor-related *α*, FoxO1, hepatocyte nuclear factor 4*α*, and nuclear respiratory factor 1 [30]. Our previous studies have shown that aldosterone-induced podocyte damage can be blocked by PPAR*γ*- PGC-1*α* dependent pathway [14, 31]. So further studies are required to investigate the mechanisms of Huaier in restoring PGC-1*α* expression and PGC-1*α* in regulating mitochondrial function.

Several groups reported some interesting mitochondria-targeted molecular pathways in podocytes including sirtun1-PGC-1*α* axis [14, 32], Rho-associated, coiled-coil-containing protein kinase 1 [12], and mTOR. But none of them have been applied in clinical practice. Clinically, other therapies targeting oxidative stress attenuation in podocytes include using the radical scavenger edaravone [33] and administering the antioxidants probucol and vitamin E [34]. Except for vitamin E [35], the other two agents are still in the animal-experiment phase [33, 34]. Compared with these novel therapies, Huaier has irreplaceable advantages by virtue of its 1,600-year history of safe and effective clinical use [36]. In the past, Huaier was often administered to patients who are easy to get infection. Recently it has been recognized clinically that Huaier also helps to ease the proteinuria, especially for children with minimal change disease. To date, no obvious side effect was observed or reported. Based on our studies, Huaier could be a promising podocyte-targeted therapy. We are investigating the underlying pharmacological mechanisms of Huaier to more rigorously assess it for clinical therapy. Additionally, our center has joined in a double-center, prospective, randomized, and double-blinded clinical trial of Huaier with Children's Hospital of Fudan University in Shanghai. The clinical efficacy and safety of Huaier will be further evaluated.

Intriguingly, Huaier has also been applied in antitumor treatments as a complementary therapy. Wang et al. demonstrated that Huaier extract dose-dependently arrests the cell cycle, promotes apoptosis, and decreases the levels of signaling molecules, such as phosphorylated extracellular signal-regulated kinase and c-Jun N-terminal kinase. This effect is significant at a dose greater than 2 mg/mL* in vitro* and 2.5 g/kg* in vivo* [36]. Zhang et al. reported that a high dose of Huaier (4–8 mg/mL) decreases MMP and consequently induces apoptosis in a breast cancer cell line [37]. These results do not conflict with our data because the dose of Huaier we administered was much lower (0.2 mg/mL* in vitro* and 2 mg/kg* in vivo*). To our knowledge, this is the first report that different doses of Huaier have distinct pharmacological effects. Relatively lower doses of Huaier tend to protect mitochondria, while high doses induce mitochondrial damage in cancer cells. The underlying mechanism is still unclear, waiting to be discovered.

In conclusion, our investigation indicates that Huaier protects podocytes against ADR-induced cytotoxicity (which mimics the “podocytopathies” of FSGS) by reversing mitochondrial dysfunction, most likely by maintaining the expression of PGC-1*α* and its downstream molecules. Huaier might be a promising alternative therapy for nephrotic syndrome.

## Figures and Tables

**Table 1:** Primer sequences of rat for real-time RT-PCR.

Gene (rat)	Forward (5′-3′)	Reverse (3′-5′)
Nephrin	ACAGCAGCCTCTTGACCAT	TGACAACCTTCAGTCCCAGT
Podocin	CAGCCACGGTAGTGAATGGG	TCAGGGAGGAGAGGACAAGA
GAPDH	CAAGTTCAACGGCACAGTCAA	TGGTGAAGACGCCAGTAGACTC
PGC-1*α*	TCAGCGGTCTTAGCACTCA	TCTCTGTGGGTTTGGTGTGA
TFAM	AAGGTGTATGAAGCGGATTTT	CGAGGTCTTTTTGGTTTTCC
mtDNA	ATCCTCCCAGGATTTGGAAT	ACCGGTAGGAATTGCGATAA
18S rRNA	TTCGGAACTGAGGCCATGATT	TTTCGCTCTGGTCCGTCTTG

**Table 2:** Primer sequences of mouse for real-time RT-PCR.

Gene (mouse)	Forward (5′-3′)	Reverse (3′-5′)
Nephrin	CCCAGGTACACAGAGCACAA	CTCACGCTCACAACCTTCAG
Podocin	GTGAGGAGGGCACGGAAG	AGGGAGGCGAGGACAAGA
GAPDH	GTCTTCACTACCATGGAGAAGG	TCATGGATGACCTTGGCCAG
PGC-1*α*	CGGAAATCATATCCAACCAG	TGAGGACCGCTAGCAAGTTTG
mtDNA	TTTTATCTGCATCTGAGTTTAATCCTGT	CCACTTCATCTTACCATTTATTATCGC
18S rRNA	GGACCTGGAACTGGCAACAT	GCCCTGAACTCTTTTGTGAAG
